# Risk Prediction Model for Severe Postoperative Complication in Bariatric Surgery

**DOI:** 10.1007/s11695-017-3099-2

**Published:** 2018-01-12

**Authors:** Erik Stenberg, Yang Cao, Eva Szabo, Erik Näslund, Ingmar Näslund, Johan Ottosson

**Affiliations:** 1 Department of Surgery, Faculty of Medicine and Health, Örebro University, Örebro, Sweden; 2 Department of Surgery, Örebro University Hospital, SE-70185 Örebro, Sweden; 3 Clinical Epidemiology and Biostatistics, School of Medical Sciences, Örebro University, Örebro, Sweden; 4 Unit of Biostatistics, Institute of Environmental Medicine, Karolinska Institutet, Stockholm, Sweden; 5 Division of Surgery, Department of Clinical Sciences, Danderyd Hospital, Karolinska Institutet, Stockholm, Sweden

**Keywords:** Morbid obesity, Prediction model, Postoperative complication, Bariatric surgery, Risk factor

## Abstract

**Background:**

Factors associated with risk for adverse outcome are important considerations in the preoperative assessment of patients for bariatric surgery. As yet, prediction models based on preoperative risk factors have not been able to predict adverse outcome sufficiently.

**Objective:**

This study aimed to identify preoperative risk factors and to construct a risk prediction model based on these.

**Methods:**

Patients who underwent a bariatric surgical procedure in Sweden between 2010 and 2014 were identified from the Scandinavian Obesity Surgery Registry (SOReg). Associations between preoperative potential risk factors and severe postoperative complications were analysed using a logistic regression model. A multivariate model for risk prediction was created and validated in the SOReg for patients who underwent bariatric surgery in Sweden, 2015.

**Results:**

Revision surgery (standardized OR 1.19, 95% confidence interval (CI) 1.14–0.24, *p* < 0.001), age (standardized OR 1.10, 95%CI 1.03–1.17, *p* = 0.007), low body mass index (standardized OR 0.89, 95%CI 0.82–0.98, *p* = 0.012), operation year (standardized OR 0.91, 95%CI 0.85–0.97, *p* = 0.003), waist circumference (standardized OR 1.09, 95%CI 1.00–1.19, *p* = 0.059), and dyspepsia/GERD (standardized OR 1.08, 95%CI 1.02–1.15, *p* = 0.007) were all associated with risk for severe postoperative complication and were included in the risk prediction model. Despite high specificity, the sensitivity of the model was low.

**Conclusion:**

Revision surgery, high age, low BMI, large waist circumference, and dyspepsia/GERD were associated with an increased risk for severe postoperative complication. The prediction model based on these factors, however, had a sensitivity that was too low to predict risk in the individual patient case.

## Introduction

Bariatric surgery is currently the only treatment available for morbid obesity that has been shown to offer significant weight loss over time [1], reduce cardiovascular morbidity [2], diabetes [3–5], cancer [6], and improve quality-of-life [7–9]. Each year, approximately 500,000 bariatric procedures are performed worldwide [10]. In the preoperative evaluation of the patient, it is important to identify factors that may be associated with adverse outcome. A few risk prediction models for postoperative mortality have been described previously [11, 12], but mortality rates following bariatric surgery today are so low that it is more important to consider severe postoperative morbidity when assessing adverse outcome in the early postoperative period [13–15]. Prediction models for postoperative complication could also enable case-mix comparison of results from different bariatric surgical centres. Risk prediction models for adverse postoperative outcome previously reported in the literature [14, 16, 17] have shown discriminatory values that are too low to be clinically useful in the individual assessment of patients [18].

The purpose of the present study was to construct and validate a risk prediction model using preoperatively available risk factors for severe postoperative complications.

## Methods

### Design and Data Sources

The Scandinavian Obesity Surgery Registry (SOReg) is a Swedish national quality and research register, and since 2010, it has covered virtually all bariatric surgical procedures performed in Sweden. The register has been described in detail elsewhere [13, 19]. Patients registered in the SOReg between 2010 and 2015 were included in the present study. All patients who underwent a bariatric procedure between 2010 and 2014 were included in the construction of the risk calculation model. Data from patients who underwent a bariatric surgical procedure in 2015 were used to validate the model’s performance in predicting serious postoperative complication within 30 days after surgery.

Historically, most bariatric procedures performed in Sweden are laparoscopic gastric bypass [13]. The surgical technique for this procedure is well standardized in Sweden with 99% being the antecolic, antegastric laparoscopic Roux-en-Y gastric bypass procedure described by Lönroth and Olbers [20]. Pharmacological prophylaxis for deep venous thrombosis is given on a routine basis [21].

Comorbidity was defined as a medical condition requiring ongoing pharmacological or positive airway pressure treatment (in the case of sleep apnoea). Previous venous thromboembolism was defined as previous treatment for deep venous thrombosis or pulmonary embolism. Smoking was defined as active smoking or a history of smoking.

### Outcome

The primary outcome measure was severe postoperative complication occurring within the first 30 days after surgery. This was defined according to the Clavien-Dindo classification of postoperative complications [22]. A complication graded as IIIb–V on this scale, i.e. a complication requiring intervention under general anaesthesia, or resulting in organ failure or death of the patient, was considered to be a severe complication.

### Data Analysis

Baseline data for the study group and the validation group are presented as number of individuals (*n*) and percentage of patients (%) for categorical values, and mean ± standard deviation (SD) for continuous variables. A logistic regression model was used to examine associations between potential risk factors and severe complications, measured as odds ratio (OR) and corresponding 95% confidence interval (CI). Continuous factors with skewed distribution were analysed on a logarithmic scale to approximate normal distribution.

Predetermined potential risk factors were examined in both univariate and multivariate analyses. All risk factors with a *p* < 0.10 in the multivariate analyses were then included in the design of a concise risk prediction model. Standardized coefficients were used to assess relative risk contribution of these factors. The standardized coefficient (ß*) was calculated from the original coefficient (ß) divided by the standard deviation of the corresponding explanatory variable. These standardized coefficients (ß*s) were then used to calculate a standardized OR for each factor and its contribution to the risk for severe complication.

Model discrimination values were evaluated using data from all patients operated during 2015 and success defined by the following: non-significance in the Hosmer and Lemeshow goodness of fit test, Nagelkerke *R*^2^ ≥ 0.10, and an area ≥ 0.7 under the receiver operating characteristic (ROC) curve.

All analyses were performed with SPSS version 22 (IBM Corporation, Armonk, New York, USA) and Stata version 14 (Stata Corp LP, College Station, Texas, USA).

### Ethics

The study was approved by the Regional Ethics Committee in Stockholm and was conducted in accordance with the ethical standards of the Helsinki Declaration (6th revision).

## Results

A total of 37,811 patients operated between January 1, 2010 and December 31, 2014 were identified in the SOReg. These patients were included in the study group. Follow-up at day 30 was 98.2% (*n* = 37,134). A further 6250 patients operated between January 1, 2015 and December 31, 2015 constituted the validation group. In this group, follow-up at day 30 was 94.7% (*n* = 5919).

The two groups were comparable with regard to baseline characteristics (Table 1). The most common primary bariatric procedure in the study group was gastric bypass (*n* = 34,161, 90.3%), followed by sleeve gastrectomy (*n* = 1774, 4.7%), duodenal switch (*n* = 252, 0.7%), gastric banding (*n* = 111, 0.3%), and other procedures (*n* = 146, 0.4%). In addition, 1367 patients (3.6%) underwent revision surgery. Most procedures were laparoscopic (*n* = 36,539, 96.6%), while 1037 patients (2.7%) underwent a planned open procedure and 235 patients (0.6%) were converted to open surgery. In the study group, 3116 patients (8.4%) suffered from some form of complication. A severe postoperative complication occurred in 1220 cases (3.3%).

**Table 1:** Baseline characteristics

	Study group		Validation group	
	Operated in 2010–2014	Missing data, *n*	Operated in 2015	Missing data, *n*
Individuals, *n*	37,811		6250	
Sex		0		0
Male, *n* (%)	9129 (24.1%)		1418 (22.7%)	
Female, *n* (%)	28,682 (75.9%)		4832 (77.3%)	
Age at operation (mean ± SD), years	41.2 ± 11.2	0	41.2 ± 11.5	0
Comorbidity, *n* (%)	20,222 (53.5%)	0	3576 (57.2%)	0
Sleep apnoea, *n* (%)	3792 (10.0%)	0	622 (9.9%)	0
Hypertension, *n* (%)	9760 (25.8%)	0	1563 (25.0%)	0
Diabetes, *n* (%)	5407 (14.3%)	0	761 (12.2%)	0
Dyslipidaemia, *n* (%)	3802 (10.1%)	0	518 (8.3%)	0
Dyspepsia/GERD, *n* (%)	3970 (10.5%)	0	645 (10.3%)	0
Depression, *n* (%)	5609 (14.8%)	0	1096 (17.5%)	0
Musculoskeletal pain, *n* (%)	4905 (13.0%)	0	1315 (21.0%)	0
Other condition, *n* (%)	4826 (12.8%)	248	465 (7.4%)	5
Previous venous thromboembolism, *n* (%)	918 (2.4%)	3645	182 (2.9%)	14
Smoking		6452		780
Non	21,484 (68.5%)		3773 (69.0%)	
Active smoking	4844 (15.4%)		798 (12.8%)	
Previous history of smoking	5031 (13.3%)		899 (16.4%)	
BMI (mean ± SD), kg/m^2^	42.12 ± 5.66	0	41.22 ± 5.87	0
Waist circumference (mean ± SD), cm	126.0 ± 13.99	6757	123.3 ± 14.05	1080
HbA1c	41.18 ± 12.52	6584	39.48 ± 10.86	991

In the validation group, 4403 patients (70.4%) were operated with gastric bypass, 1696 (27.1%) with sleeve gastrectomy, 55 (0.9%) with duodenal switch, 25 (0.6%) with another bariatric procedure, and 71 patients (1.1%) underwent revision surgery. Most procedures were laparoscopic (*n* = 6129, 98.1%), while 98 patients (1.6%) underwent a planned open procedure and 23 patients (0.4%) were converted to open surgery. In the validation group, 482 patients (8.1%) suffered from some form of postoperative complication. A severe postoperative complication occurred in 188 cases (3.2%). Potential risk factors for severe postoperative complication analysed in the study group are presented in Table 2.

**Table 2:** Risk for severe postoperative complication

	Unadjusted analyses			Adjusted analyses	
	*n* (%)	OR (95%CI)	*P*	OR (95%CI)	*P*
Sex					
Men	304 (3.4%)	Reference	Reference	Reference	Reference
Women	916 (3.3%)	0.96 (0.84–1.10)	0.549	1.07 (0.88–1.30)	0.491
Age				1.01 (1.00–1.02)	0.009
< 30 years	161 (2.5%)	Reference	Reference		
30–40 years	283 (3.0%)	1.18 (0.97–1.44)	0.093		
40–50 years	424 (3.5%)	1.41 (1.18–1.70)	< 0.001		
50–60 years	289 (3.9%)	1.58 (1.30–1.92)	< 0.001		
> 60 years	63 (3.7%)	1.47 (1.09–1.98)	0.011		
Waist circumference				1.01 (1.00–1.02)	0.070
< 110 cm	92 (2.8%)	Reference	Reference		
110–125 cm	360 (3.2%)	1.15 (0.91–1.45)	0.235		
125–140 cm	358 (3.3%)	1.18 (0.94–1.49)	0.158		
> 140 cm	152 (3.1%)	1.11 (0.86–1.45)	0.419		
Body mass index				0.98 (0.96–1.00)	0.019
< 40 kg/m^2^	496 (3.5%)	Reference	Reference		
40–50 kg/m^2^	630 (3.2%)	0.93 (0.82–1.05)	0.220		
50–60 kg/m^2^	84 (2.9%)	0.83 (0.66–1.05)	0.116		
> 60 kg/m^2^	10 (2.9%)	0.82 (0.43–1.54)	0.534		
Ln HbA1c		1.32 (1.03–1.70)	0.031	1.07 (0.71–1.61)	0.751
Comorbidity					
Sleep apnoea	136 (3.6%)	1.12 (0.94–1.35)	0.208	0.95 (0.74–1.22)	0.659
Hypertension	356 (3.7%)	1.18 (1.04–1.34)	0.010	0.96 (0.80–1.16)	0.705
Diabetes	203 (3.8%)	1.19 (1.02–1.39)	0.026	1.05 (0.80–1.38)	0.705
Dyslipidaemia	135 (3.6%)	1.10 (0.92–1.32)	0.298	0.81 (0.62–1.05)	0.105
Dyspepsia/GERD	167 (4.3%)	1.36 (1.15–1.60)	< 0.001	1.25 (1.02–1.54)	0.034
Depression	200 (3.6%)	1.15 (0.97–1.32)	0.113	1.15 (0.95–1.39)	0.152
Musculoskeletal pain	151 (3.1%)	0.95 (0.80–1.13)	0.535	0.90 (0.69–1.19)	0.471
Other comorbidity	178 (3.7%)	1.16 (0.99–1.36)	0.075	1.02 (0.78–1.33)	0.887
Previous venous thromboembolism	43 (4.8%)	1.52 (1.11–2.07)	0.009	1.29 (0.87–1.92)	0.205
History of smoking	322 (3.3%)	1.04 (0.91–1.19)	0.558		
Surgical year				0.95 (0.90–1.01)	0.083
2010	276 (3.7%)	Reference	Reference		
2011	279 (3.4%)	0.90 (0.76–1.06)	0.215		
2012	255 (3.4%)	0.91 (0.77–1.08)	0.293		
2013	241 (3.2%)	0.86 (0.72–1.02)	0.091		
2014	169 (2.6%)	0.69 (0.57–0.83)	< 0.001		
Surgical access					
Laparoscopic	1123 (3.1%)	Reference	Reference	Reference	Reference
Open	68 (6.7%)	2.21 (1.72–2.85)	< 0.001	1.38 (0.89–2.15)	0.147
Conversion	29 (12.5%)	4.42 (2.98–6.55)	< 0.001	3.22 (1.89–5.51)	< 0.001
Operation method					
Gastric bypass	1071 (3.2%)	Reference	Reference	Reference	reference
Sleeve gastrectomy	29 (1.7%)	0.53 (0.37–0.77)	0.001	0.72 (0.48–1.06)	0.096
Duodenal switch	8 (3.2%)	1.00 (0.50–2.04)	0.989	1.38 (0.33–5.77)	0.658
Gastric banding	1 (0.9%)	0.28 (0.04–2.00)	0.204	0.72 (0.10–5.25)	0.747
Other	5 (4.3%)	1.36 (0.55–3.33)	0.505	1.26 (0.46–3.45)	0.649
Revisional surgery	106 (7.9%)	2.68 (2.18–3.30)	< 0.001	2.27 (1.65–3.10)	< 0.001

The factors included in the risk prediction model are presented in Table 3. The largest contribution came from revision surgery (19.0%), followed by low body mass index (10.8%), age (9.7%), year of surgery (9.5%), waist circumference (8.8%), and dyspepsia/GERD (8.2%). When the model was recalculated using only primary gastric bypass procedures, no significant difference was found (data not shown).

**Table 3:** Adjusted risk for severe postoperative complication based on standardised parameters

	Full model			Concise model		
	Contribution to risk			Contribution to risk		
	OR (95%CI)	*P*	%	OR (95%CI)	*P*	%
Female sex	1.03 (0.95–1.12)	0.520	2.77			
Age	1.12 (1.03–1.22)	0.008	12.24	1.10 (1.03–1.17)	0.007	9.70
Waist circumference	1.10 (0.99–1.23)	0.078	10.35	1.09 (1.00–1.19)	0.059	8.81
Body mass index	0.99 (0.89–1.09)	0.036	1.08	0.89 (0.82–0.98)	0.012	10.76
Ln HbA1c	1.02 (0.98–1.06)	0.700	1.95			
Coexisting medical condition						
Sleep apnoea	0.98 (0.95–1.02)	0.661	1.65			
Hypertension	1.01 (0.96–1.01)	0.727	1.46			
Diabetes	1.02 (0.98–1.07)	0.652	2.36			
Dyslipidaemia	0.94 (0.83–1.06)	0.120	6.14			
Dyspepsia/GERD	1.08 (0.93–1.06)	0.029	7.65	1.08 (1.02–1.15)	0.007	8.21
Depression	1.05 (0.98–1.12)	0.176	3.30			
Musculoskeletal pain	0.97 (0.90–1.04)	0.381	3.30			
Previous venous thromboembolism	1.04 (0.98–1.11)	0.190	4.25			
Surgical year	0.92 (0.85–0.98)	0.016	8.42	0.91 (0.85–0.97)	0.003	9.49
Revisional surgery	1.19 (1.14–1.25)	< 0.001	19.23	1.19 (1.14–1.24)	< 0.001	18.96

**Fig. 1:**
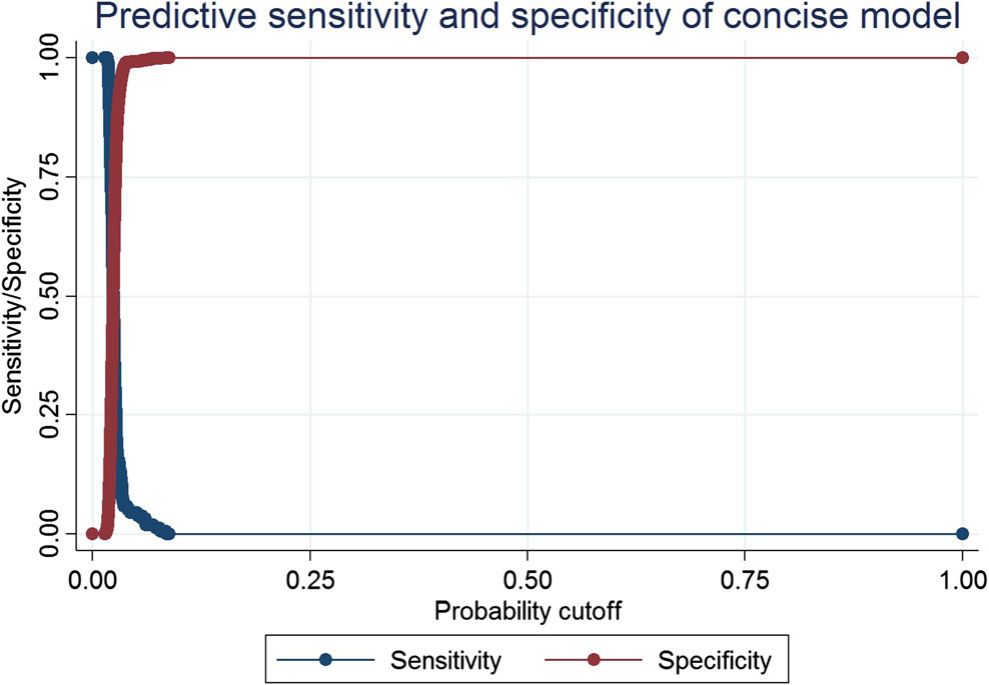
Sensitivity/specificity graph for the risk calculation model based on data from patients operated within the validation group

Validation of the model tested on patients operated in 2015 resulted in an area under the ROC curve of 0.53, a Hosmer-Lemeshow goodness of fit 17.91 (*p* = 0.056) and Nagelkerke R2 0.013. The sensitivity/specificity graph is presented in Fig. 1.

**Fig. 2:**
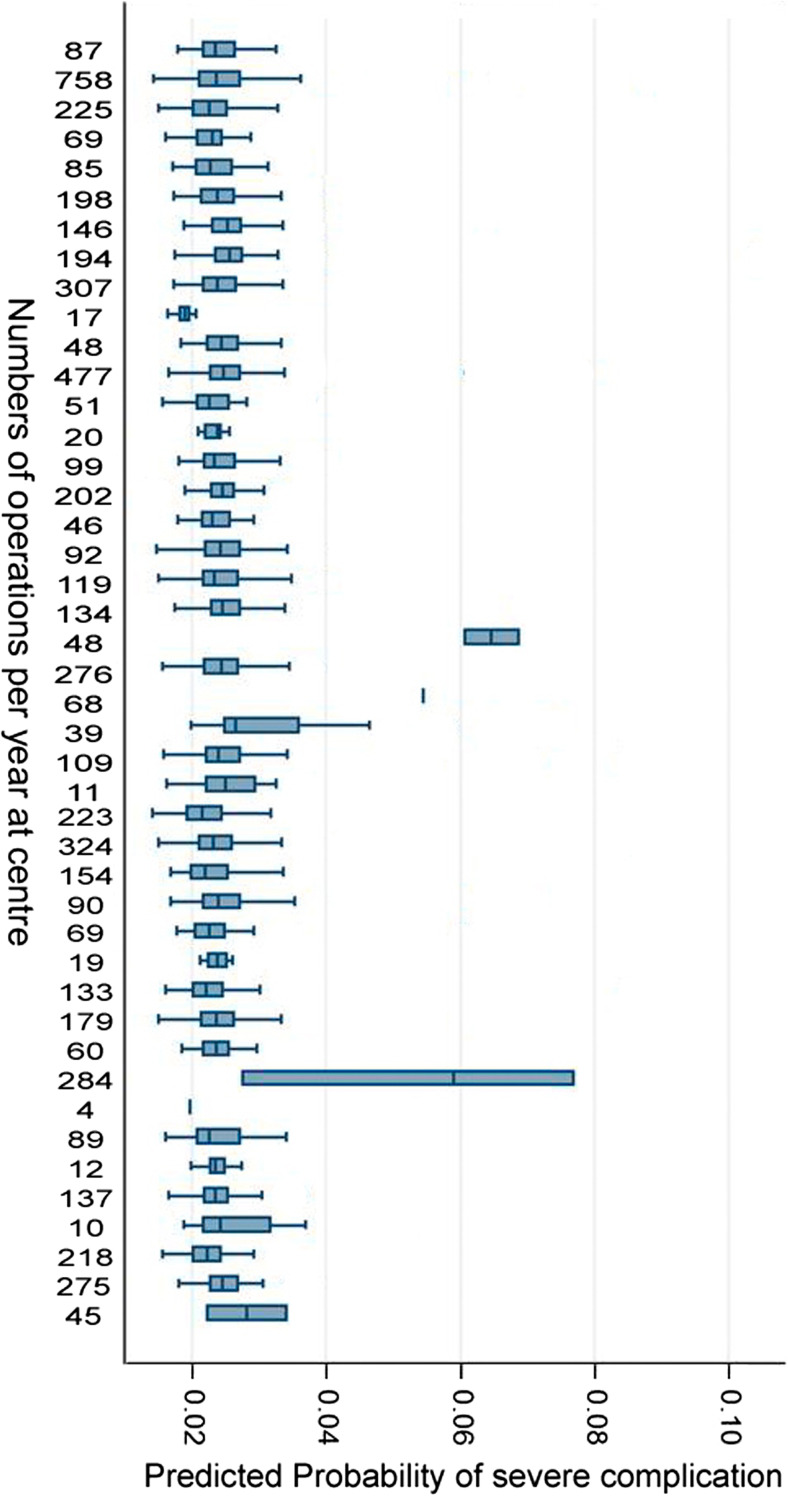
Predicted probability of severe complication per centre for patients operated in 2015

The differences in case-mix, estimated as predicted probability of severe postoperative complication, between different centres during 2015 is presented in Fig. 2.

## Discussion

In the present study, a number of factors known at the time of surgery were identified as being associated with increased risk for severe postoperative complication. In general, the risk prediction model based on these factors had a high specificity, but a low sensitivity. Despite the large study population, it was not possible to create a prediction model that was sensitive enough to be useful for individual patients in clinical practice. Indeed, surgery-specific risk factors seem to be the most important factors when predicting severe postoperative complications [13, 18]. The present study supports the results of a review by Geubbels reporting that risk prediction models based on preoperatively known risk factors alone are inadequate in predicting postoperative severe complications [18]. There are, however, a number of preoperative factors associated with increased risk for severe postoperative complication.

In the present study, high age, dyspepsia/GERD, relatively low BMI, large waist circumference, and revision surgery were associated with increased risk for severe postoperative complication within 30 days after surgery. Age has previously been reported to be an important patient-specific risk factor for severe postoperative complication [13, 14, 17, 18]. An association between lower BMI and increased risk for postoperative complication has also been reported previously [13, 16]. The subgroup of patients with a relatively low BMI accepted for surgery may have a different metabolic profile than the average bariatric surgical patient. The combination of large waist circumference and a relatively low BMI indicates a fat distribution associated with higher metabolic burden [23, 24]. This could also be associated with increased insulin resistance as a response to the surgical trauma, or simply the cause of more technically demanding surgery. Insulin resistance has previously been suggested as a factor associated with postoperative complications [25, 26]. Dyspepsia/GERD may increase the risk for severe pulmonary complications following surgery [27]. In the present study, an association between dyspepsia/GERD and severe postoperative complication was also seen, and likely explained by the increased risk for postoperative gastric and pulmonary complications. However, dyspepsia/GERD may also serve as a confounder for other risk factors, such as psychosomatic disorders, not measured within this study [28–30]. Revision surgery is more technically demanding than primary surgery and complication rates are also known to be higher [31]. Finally, our model included year of surgery. This factor is not relevant when considering risk in a specific case, but since complication rates improve with time, it is an important factor to consider when designing a prediction model.

This study was based on data from almost all bariatric surgery patients operated in Sweden during the study period. Follow-up at 30 days after surgery was also high. There are, however, a number of limitations that must be acknowledged. HbA1c did not fit in the present risk prediction model. Although an association between HbA1c and risk has been established in diabetic non-obese subjects undergoing cardiovascular surgery [26], for morbidly obese patients undergoing bariatric surgery the association has only been established in non-diabetic patients [25]. Since our aim was to create one model for all bariatric surgery patients, both diabetic and non-diabetic patients were grouped together, though it is possible that risk association is somewhat different in diabetic compared to non-diabetic morbidly obese patients. A model incorporating several potential predictor variables may have multicollinearity problems. However, in our model, multicollinearity did not affect how well the model fitted. The study was a register-based observational study. A register-based study is limited to the specific definitions of the register and to the quality of registration. Registration in the SOReg is subjected to continuous validation, and so far, the validity has been shown to be very high [19]. Cardiovascular comorbidity and pulmonary comorbidity other than sleep apnea are not mandatory variables in the SOReg and could thus not be evaluated as potential risk factors in this study. Although cardiovascular disease is over-represented in obese patients [32], the prevalence of severe cardiovascular comorbidity in this group of patients at the time of surgery is not that high in European studies [18]. Furthermore, patient-specific socio-economic factors such as level of education, employment/unemployment, married or not, or immigration, could play an important role. Such data were not available for this study as they are not entered in the SOReg.

Although prediction models based on preoperative factors have not been found to be useful in the preoperative assessment of patients prior to bariatric surgery, the results of the present study indicate that dyspepsia/GERD, high age, and large waist circumference relative to BMI (signalling significant amount of visceral fat) should be considered as risk factors in the individual case, in particular patients considered for revision surgery. To optimize these patients, pharmacological treatment for dyspepsia/GERD and a preoperative weight loss of 5–10% TBW [33] may be considered.

Although our prediction model had too low a sensitivity for use at the individual level, the specificity was very high and our results could be used for case-mix adjustments on a group level. This could be useful when comparing results between different units or in reimbursement systems. For prediction, classification and pattern recognition purposes, when traditional statistical modelling methods (dealing with finding relationship between variables to predict an outcome) do not work, machine learning methods (which deals with building systems that can learn from data, instead of explicitly programmed instructions) may be promising alternatives.

### Conclusion

Revision surgery, age, low BMI, waist circumference, and dyspepsia/GERD were associated with a higher risk for severe postoperative complication. However, a prediction model based on these factors, despite its high specificity, had a low sensitivity for severe postoperative complications.
